# Seasonal Variability of the Biochemical Composition and Antioxidant Properties of *Fucus spiralis* at Two Azorean Islands

**DOI:** 10.3390/md16080248

**Published:** 2018-07-26

**Authors:** Lisete Paiva, Elisabete Lima, Ana Isabel Neto, José Baptista

**Affiliations:** 1 Biotechnology Centre of Azores (CBA), University of Azores, 9501-801 Ponta Delgada, São Miguel, Azores, Portugal; elisabete.mc.lima@uac.pt (E.L.); jose.ab.baptista@uac.pt (J.B.); 2 Institute of Agricultural and Environmental Research and Technology (IITAA), University of Azores, 9501-801 Ponta Delgada, São Miguel, Azores, Portugal; 3 Azorean Biodiversity Group (ABG), Centre for Ecology, Evolution and Environmental Changes (cE3c), Department of Biology, University of Azores, 9501-801 Ponta Delgada, São Miguel, Azores, Portugal; ana.im.neto@uac.pt

**Keywords:** marine functional foods, antioxidant activities, brown algal phlorotannins, phenolic and flavonoid contents, protein, dietary fiber, carbohydrates, lipids, fatty acids profile, energy value

## Abstract

This study evaluates, for the first time, the seasonal (winter and summer) and geographical (São Miguel–SMG and Santa Maria–SMA Islands) variability of *Fucus spiralis* (Fs) biochemical composition (dry weight basis) and antioxidant properties. Protein and carbohydrates presented higher values in Fs-SMG_winter_, lipids, total dietary fiber, and energy value in Fs-SMA_summer_, and ash and soluble dietary fiber/insoluble dietary fiber ratio in Fs-SMA_winter_. The fatty acid (FA) profiles showed a lower SFA in Fs-SMG_summer_, whereas MUFA and PUFA presented higher values in Fs-SMG_summer_ and Fs-SMG_winter_, respectively. Excellent dietary ratios of *n*6/*n*3 PUFA and hypocholesterolemic/hypercholesterolemic FA were found, with lower values in Fs-SMA_winter_ and higher in Fs-SMG_summer_, respectively. The highest total phenolics was found in Fs-SMA_summer_ acetone:water extract and total flavonoids showed the higher value in Fs-SMG_winter_ methanol extract. The best free radical-scavenging activity was observed in the Fs-SMA_winter_ methanol (EC_50_ = 0.045 mg/mL) and acetone:water (EC_50_ = 0.059 mg/mL) extracts. The ferric-reducing antioxidant power showed the best results in Fs-SMA_winter_ methanol extract (EC_50_ = 0.016 mg/mL) and Fs-SMA_summer_ acetone:water extract (EC_50_ = 0.017 mg/mL). The best ferrous ion-chelating activity was found in Fs-SMG_winter_ acetone:water extract. Overall, results revealed that *F. spiralis* nutritional and functional bioactivity values have geographical and seasonal variations and that its regular consumption may add benefits to human health.

## 1. Introduction

The ever-increasing human population around the world has led to a constant search for new resources to meet the growing demand for food and medicine. The marine ecosystems, particularly the macroalgae (commonly referred to as “seaweeds”), appear to be a valuable natural resource for these needs. Indeed, besides their nutritional value, seaweeds are an excellent source of health-promoting metabolites due to their living mode in highly competitive and aggressive environments, which are very different in many aspects from the terrestrial ones. Such situations demand the production of quite specific and potent bioactive molecules, which may lead to the development of new drugs and functional foods or nutraceuticals. In fact, seaweeds are increasingly being recognized as a rich natural resource of valuable biochemical constituents (including high-quality proteins, bioactive peptides, dietary fiber, polysaccharides, lipids, fatty acids, minerals, vitamins, pigments, and phytochemicals such as polyphenols), that may have protective effects against allergy, cancers, degenerative disorders, diabetes, digestive disorders, heart diseases, hypertension, inflammation, lipidemia, obesity, and oxidative stress [1,2,3,4,5,6]. However, as is well established, the biochemical composition of seaweeds, and consequently their nutritional and medicinal values, depends on many factors, such as: species and its developmental stage, spatial and temporal changes in environmental parameters (including light, temperature, nutrients, and salinity) and biotic interactions [1,6,7,8,9,10,11].

The Azores Islands (Portugal), being isolated in the middle of Atlantic Ocean (37°40′ N and 25°31′ W) and surrounded by seawater with low pollution levels [12], are a very promising location to look for new marine metabolites with health promoting effects in treating/preventing of chronic diseases and/or for use in food industries. Traditionally, the Azorean population has gathered seaweeds to use as fertilizers in local agriculture and/or horticulture, and to eat (e.g., *Fucus spiralis*, *Porphyra*, *Laurencia*, *Osmundea*, and *Ulva*) or for agar production (e.g., *Gelidium microdon* and *Pterocladiella capillacea*). Namely, the brown seaweed *F. spiralis*, which is common in the Azorean intertidal zone, is a local delicacy, particularly the frond tips (the receptacles) that are picked and eaten fresh [13]. Previous studies on its nutritional and/or pharmacological value from our research group have reported that *F. spiralis* is a good source of valuable biochemical compounds [14] with potential impact on human health [15,16], particularly the bioactive phlorotannins that are the predominant polyphenols in brown algae, being abundant in Fucaceae [17]. *F. spiralis* from other origins have also been demonstrated to possess some important biological activities, such as high antioxidant properties that are mainly linked to its phlorotannins content [18,19,20].

To the best of our knowledge, this is the first study investigating the seasonal (winter and summer) and geographical (São Miguel–SMG and Santa Maria–SMA Islands) variations in the biochemical composition and antioxidant properties of the Azorean *F. spiralis* as a source of functional products with multi-bioactivities for potential use in the food and pharmaceutical industries. This study was aimed to: (i) determine the biochemical composition (protein, lipids, fatty acids, soluble carbohydrates, ash, energy value, and total, soluble, and insoluble dietary fiber) of the four different *F. spiralis* samples; (ii) determine the total phenolic and total flavonoid contents of the methanol and acetone:water (7:3) extracts from the four different *F. spiralis* samples; and (iii) evaluate the *F. spiralis* antioxidant properties of the referred extracts using different and significant assays (scavenging of the 2,2-diphenyl-1-picrylhydrazyl free radical, ferrous ion-chelating activity, and ferric reducing antioxidant power) in order to accurately reflect the in vivo complexity of interactions between antioxidants of the algal extracts.

## 2. Results and Discussion

### 2.1. Crude Protein Determination

The protein content of macroalgae varies between 3% and 47% on a dry weight (DW) basis, depending to a large extent on the phylum and species [1]. According to this author, generally the green and red algae contain higher protein levels (10–47% DW) than brown algae (3–15% DW). In addition, the protein content varies with geographical location and seasonal conditions, being highly influenced by seawater temperature, salinity, and nutrients [1,21,22,23]. The protein content (DW) of *F. spiralis* (Table 1) ranged from 4.14% to 8.25% for SMA and SMG summer samples, respectively, and from 6.85% to 9.71% for winter samples of the same locations, respectively, and presented significant differences (*p* < 0.05) among the four samples. The results revealed that *F. spiralis* protein content is within the range reported for brown algae [1] and presented higher values in SMG than in SMA, being the best results achieved in winter season. The results clearly show the influence of seasonal conditions on *F. spiralis* protein content that can be explained by lower seawater temperature and salinity in winter than in summer season in both locations (see Section 3.2). Rupérez and Saura-Calixto [24] described similar results for *Fucus vesiculosus* (6.19% DW) collected in Pontevedra (Spain). Furthermore, Rodrigues [25] also reported a higher value (13.2% DW) for the same species collected in winter season in Aveiro (Portugal). Zavodnik [7] also reported a maximum value in winter (12.3% DW) and a minimum in summer (5.1% DW) for *Fucus virsoides*.

On the other hand, the bioavailability of algal proteins can sometimes be inhibited by the entrapped nature of the proteins in the cellular matrix. Increasing the bioavailability by using physical processes or fermentation to break down the fibers and release the protein has been investigated [26]. Overall, macroalgae have been favorably reviewed as sources of proteins for nutritional purposes [27]. Furthermore, according to Moskaug et al. [28] macroalgae may be able to regulate glutathione synthesis with their high polyphenol levels, which have been recently linked to increased expression of glutamylcysteine.

### 2.2. Soluble Carbohydrate Content Determination

In some studies, the carbohydrate content was calculated by subtracting the protein, lipid, ash, and moisture from 100%. This study measured soluble carbohydrates colorimetrically, and found concentrations in a narrow range as shown in Table 1. The highest values were observed in SMG winter (17.59% DW) followed by SMA summer (17.03% DW), whereas the lowest values were recorded in SMG summer (13.45% DW) and SMA winter (12.77% DW) with significant differences (*p* < 0.05) between seasons in both locations. Similar results were reported by Chakraborty and Santra [29] for the brown alga *Dictyota ceylanica* (18.52% DW), and by Manivannan et al. [30] that also found concentrations in a narrow range of 21.88–23.90% of DW for several brown algae from Mandapam, southeast coast of India.

According to Rosemberg and Ramus [31] the algae carbohydrate synthesis is related to periods of maximum growth, increased photosynthetic activity and a reduction in protein content. On the other hand, according to Munda and Kremer [21], these periods were influenced by increased values of seawater temperature, salinity, and sunlight intensity, which confirms the influence of these parameters (see Section 3.2) on carbohydrate synthesis of *F. spiralis* from SMA. Furthermore, an inverse relationship was observed in these samples between carbohydrates (higher in summer) and protein (higher in winter). A similar pattern has been reported for *F. virsoides* [7]. However, a contrary pattern was observed for *F. spiralis* from SMG that presented higher carbohydrate and protein contents in winter than in summer. Thus, these results indicate that even within the same species it is possible to find differences in the ability to synthesize carbohydrates.

### 2.3. Total Lipid Determination

The majority of marine algae have very low lipid content ranging from 0.3 to 7% of DW [32], revealing a low source of nutritional energy comparable with land vegetables [33]. The crude lipid content (DW) of the *F. spiralis* samples (Table 1) is within that range except for the SMA summer that presented a significantly higher value of 11.54%. SMG winter and summer (5.23% and 5.33%, respectively) had an intermediate position. SMA winter (4.40%) presented the minimum value and was similar to the value reported by Lorenzo et al. [34] for *F. vesiculosus* (3.75% DW). The results revealed that the lipid content from summer samples in both islands presented higher values compared to winter samples, but without significant difference (*p* < 0.05) for SMG samples. Kim et al. [35] also reported that the lipid content of the *Fucus serratus* strongly increases during summer with a value of 2.1% in August and only 0.4% in April. Similarly, Zavodnik [7] reported a maximum value in summer (6.8% DW) and a minimum in winter (2.2% DW) for *F. virsoides*. Schmid et al. [36,37] reported a similar pattern for other upper to mid-shore Fucaceae species. According to the literature [35,36,37], the higher lipid content in summer was mainly due to an accumulation of triacylglicerols as storage compounds. The significantly higher lipid content in SMA summer as compared to SMG summer is probably due to the higher seawater temperature in SMA summer season (see Section 3.2). However, according to Schmid et al. [36], although temperature represents one of the possible factors that can influence lipid content, it is likely that changes in other abiotic factors (light, salinity, and nutrients) and interactions between such factors may also contributed to the observed variations.

### 2.4. Fatty Acids (FA) Determination

According to Darcy-Vrillon [33], although the lipid content of macroalgae is relatively low, they can contain higher levels of PUFA than land vegetables. However, the macroalgae FA contents and profiles can vary with spatial and temporal differences, as well as with genetic variability [9,11,36]. Table 2 shows the FA profiles of the *F. spiralis* samples and the content of FA groups in percentage of the total fatty acids methyl esters (tFAME), on a DW basis. The saturated fatty acids (SFA) ranged from 31.01% to 40.65%, with palmitic acid (C16:0) being the most abundant SFA. The monounsaturated fatty acids (MUFA) ranged from 21.62% to 40.82% presenting the summer samples the higher values. The most dominant one was oleic acid (C18:1*n*9), ranging from 20.36% to 38.04%. The polyunsaturated fatty acids (PUFA) ranged from 28.17% to 40.59% presenting the winter samples the higher values. These results revealed an inverse relationship between MUFA (higher in summer) and PUFA (higher in winter) contents. Schmid et al. [11,36] reported a similar seasonal pattern for other upper to mid-shore Fucaceae species (*Ascophyllum nodosum*, *F. serratus*, and *F. vesiculosus*). Several authors [38] suggest that the increase in PUFA in winter could facilitate the greater cell membrane fluidity at low temperature. Among the PUFA group, the dihomo-γ-linolenic (DHGLA, C20:3*n*6) and eicosatrienoic (C20:3*n*3) acids were found in appreciable quantities, within the ranges of 9.40–13.83% and 4.33–11.31%, respectively; although these PUFA are not very common in *Phaeophyceae* species, similar values of DHGLA [39] and eicosatrienoic acid [40] were reported for same *Sargassum* species. On the other hand, the eicosapentaenoic acid (EPA, C20:5*n*3) was found within the range of 0.62–3.44% and the docosahexaenoic acid (DHA, C22:6*n*3) ranged from 1.25% to 3.80%. Furthermore, the arachidonic acid (ARA, C20:4*n*6) was found only in trace levels. A different trend on PUFA profiles was reported for other Phaeophyceae species that are particularly rich in C20 PUFA such as ARA and EPA [34,36]. In accordance with previous findings [41], these variations could be attributed to either different species, environmental factors or a combination of both.

For nutritional evaluation, the determination of the hypocholesterolemic/hypercholesterolemic (h/H) FA ratio is of fundamental importance, according to the current knowledge on the effects of specific fatty acids on cholesterol metabolism. Therefore, the higher h/H ratio, the more adequate is an oil or fat for its nutritional value [42,43]. The h/H ratio of *F. spiralis* samples presented values of 1.59, 1.89, 2.14, and 2.37 for SMA winter, SMA summer, SMG winter, and SMG summer, respectively, that are similar to those found in farmed fish species (2.03 to 2.46) [42], revealing that *F. spiralis* have a high nutritional value from the FA point of view. Furthermore, the *n*6/*n*3 FA ratio presented values of 1.51, 1.99, 2.57, and 2.94 for SMA winter, SMG winter, SMA summer, and SMG summer, respectively, that are lower than 10 as currently recommended by the WHO. According to Simopoulos [44], the increase intake of *n*6 FA in Western diets relative to *n*3 FA increase the *n*6/*n*3 ratio from the healthy value of 1:1 to 20:1 or an even higher ratio and consequently promotes the pathogenesis of many diseases, including cardiovascular disease, cancer, and inflammatory and autoimmune diseases. Therefore, the consumption of Azorean *F. spiralis* will contribute to decrease this ratio and consequently have an impact on human health. Similar results were reported by Rodrigues et al. [45] regarding the *n*6/*n*3 ratio that presented values of 1.63 and 3.09 for the edible brown algae *Saccorhiza polyschides* and *Sargassum muticum*, respectively, and by Lorenzo et al. [34] for *F. vesiculosus* (1.72). It should also be highlighted that clinical studies by Ginsberg et al. [46] have shown that diets rich in MUFA and PUFA and low in SFA reduce the total cholesterol and LDL-chol in plasma. In addition to this healthful FA profile, the content of the *trans*-FA is also low in all *F. spiralis* samples (4.35–6.15% of tFAME).

Overall, *F. spiralis* presented the highest h/H ratios in SMG samples from both seasons, as well as excellent *n*6/*n*3 ratios in the winter samples from both islands that also presented the best PUFA content, suggesting a slightly higher protective effect on inflammatory, cardiovascular, and nervous system disorders [2], as compared to SMA summer sample.

### 2.5. Total, Soluble, and Insoluble Dietary Fiber (TDF, SDF and IDS) Determination

Macroalgae are a richer source of TDF (29–62% of DW) as compared to most fruits and vegetables [4,47], with SDF and IDF accounting for 8.3–85% DW and 20–98.9% DW of TDF, respectively [32]. Indeed, SDF and IDF were highly variable between species, among other factors, although IDF is generally the dominant [32]. In addition, algae fiber differs physically and chemically from the fiber of terrestrial plants and thus induces different physiological effects [48]. As shown in Table 1, the dietary fiber was the most abundant component in all *F. spiralis* samples and the amount of TDF, SDF, and IDF ranged from 40.44% to 52.27%, 17.77% to 24.77%, and 21.69% to 27.50% of DW, respectively. The results also show higher values for SMA than for SMG samples in both seasons, and are within the ranges referred for macroalgae species. Furthermore, the *F. spiralis* from SMA presented higher TDF content than other wellknown nonmarine sources of dietary fiber, such as wheat bran (43.9% DW) [49]. Similar results were reported for TDF amount from Hawaiian Phaeophyceae species that ranged from 34.60% to 53.70% of DW [49]. According to Rupérez and Saura-Calixto [24], the *F. vesiculosus* collected in Pontevedra (Spain) presented the values of 9.80%, 40.29%, and 50.09% of DW for SDF, IDF, and TDF, respectively. Díaz Rubio et al. [50] also reported similar results from the same species collected in Galicia (Spain).

The SDF/IDF ratio of *F. spiralis* samples was 0.78, 0.86, 0.90, and 0.97 for SMG summer, SMG winter, SMA summer, and SMA winter, respectively, revealing that SDF/IDF ratio was higher in SMA than in SMG in both seasons and also higher in winter as compared to summer in each location. According to the literature, the extent to which environmental conditions or algal phenology affect the SDF and IDF contents in macroalgae is poorly documented or not known, especially from the tropical zones [49]. Similar SDF/IDF ratios were reported for other species [2] indicating that macroalgae are an excellent source of dietary fiber with surplus-value (good balance of both soluble and insoluble fibers). As a result, they may be important in the prevention and/or control of body-weight and in the prevention and/or treatment of various chronic health problems (such as gastrointestinal, cardiovascular, diabetes, and hypertension diseases) which are accordingly associated with low fiber diets of the Western countries [51]. It should also be highlighted that a study of Godard et al. [52] has demonstrated that fiber from algae prevent the fall of antioxidant defenses and the development of atherosclerosis in hamsters that were fed with a high cholesterol diet.

### 2.6. Ash Determination

According to previous studies [53,54,55], the ash content of macroalgae vary to a largely extent with species, geographical locations, and seasons. The ash content (DW) obtained in *F. spiralis* samples (Table 1) show the values of 22.43% and 29.57% for SMA summer and winter, respectively, and 22.67% and 25.40% for SMG winter and summer, respectively, which are within the ranges previously reported for marine algae and algal food products (8–44% of DW) [32,56,57]. The results also show higher values in SMA winter than in summer, and oppositely, SMG presented higher values in summer as compared to winter. In accordance with previous findings [53], these results could be explained, at least in part, by spatial and temporal fluctuations of Azorean seawater mineral content.

Rupérez [57] and Lorenzo et al. [34] reported similar values for Spanish *F. vesiculosus* from Pontevedra (30.10% DW) and A Coruña (20.71% DW) locations, respectively, and Bocanegra et al. [3] also reported the values of 19.2 to 35% of DW for Phaeophyceae algae. Furthermore, a study on seasonal variation in chemical composition of *F. virsoides* reported a maximum value in winter (23.5% DW) and a minimum in summer (14% DW) [7]. In addition, other seasonal studies on Phaeophyceae algae [58] found no overall trend in ash content, since some species had significantly higher percentages of ash during the winter and oppositely others in the summer season. Our results also revealed an inverse relationship between ash content and carbohydrates. Zavodnik [7] and Marinho-Soriano et al. [23] reported a similar pattern for *F. virsoides* and *Sargassum vulgare*, respectively.

According to Ortega-Calvo et al. [56], the ash content of macroalgae is higher than that of the most common vegetables, due to the extraordinary ability of macroalgae to accumulate minerals present in the water. Thus, the high ash content observed in *F. spiralis* can contribute with important microelements to human and animal nutrition that are rare or absent in some land vegetables.

### 2.7. Energy Value Determination

Few studies have examined the caloric content of edible seaweeds, despite its importance in human and animal nutrition. As shown in Table 1, the *F. spiralis* samples presented significantly high nutritive values in terms of high-calculated energy values: 5.43, 6.22, 7.24, and 8.12 kJ/g for SMA winter, SMG summer, SMG winter, and SMA summer, respectively. Amongst the four samples, the one from SMA summer presented higher nutritive values as compared to winter, and oppositely, SMG presented higher values in winter as compared with that in summer. Comparing both locations, SMA presented higher values in summer than SMG, and oppositely SMG presented higher values in winter as compared with summer. These results may be explained by differences in sunlight intensity, ultraviolet radiation, and salinity in both locations during the different seasons that, as previously mentioned, may account for differences in the biochemical content (protein, soluble carbohydrates, and lipids) of *F. spiralis* samples. Renaud and Luong-Van [58] reported similar calculated energy values for some Phaeophyceae algae that ranged from 4.0 to 6.4 kJ/g collected in the summer and from 4.2 to 8.6 kJ/g in the winter season.

### 2.8. Total Phenolic Content (TPC) Determination in F. spiralis Extracts

The TPC was quantified as phloroglucinol equivalents (PE), and the results, as illustrated in Table 3, are expressed in mg of PE/g of dry extract (DE), and also in mg of PE/g dry weight (DW) of algal sample just for literature comparison purposes.

*F. spiralis* from SMA shows lower values of 172 and 187 mg PE/g DE for summer and winter methanol extracts, respectively, as compared to the values of 245.67 and 243.33 mg PE/g DE for summer and winter acetone:water (7:3) extracts, respectively. *F. spiralis* from SMG also show lower values of 113 and 153.33 mg PE/g DE for summer and winter methanol extracts, respectively, as compared to the values of 229.33 and 170.67 mg PE/g DE for summer and winter acetone:water (7:3) extracts, respectively.

Regarding the extraction conditions, the results revealed that 70% aqueous acetone is a more efficient solvent for *F. spiralis* phlorotannins extraction compared to methanol, probably due to its effectiveness to release phlorotannins from protein during extraction. The results also revealed that, among the extracts under study, *F. spiralis* acetone:water extracts from SMA presented higher TPC values. Furthermore, the results also revealed that extracts from SMA presented higher values in the winter methanol extract compared with the summer one but without significant difference (*p* < 0.05) and oppositely higher values in the summer acetone:water (7:3) extract compared with the winter one but also without significant difference (*p* < 0.05). The SMG extracts showed the same behavior, however, the values from summer and winter samples presented a significant difference (*p* < 0.05) for both extracts.

The TPC values of *F. spiralis* in the present study were similar to the ones reported by Tierney et al. [59] for a Irish *F. spiralis* winter sample (22.31, 37.03, and 39.04 mg PE/g DW for water, ethanol:water and methanol:water extracts, respectively). These values also revealed the effectiveness of aqueous organic solutions on the phlorotannin extraction yield than using only water as extracting solvent.

It should also be pointed out that significant intra-species variations of TPC in brown fucoid algae are documented, showing the effect of several factors, such as: algae size, age, tissue type, locations, seasons, and environmental conditions, including nutrient availability, light intensity, ultraviolet radiation, salinity, and water depth [17,60]. However, contradictory results were found in the literature for seasonal fluctuations of TPC. In the present study, the seasonal pattern of *F. spiralis* TPC is similar. Nevertheless, current evidence suggests that the production of phlorotannins by Fucales species is tightly correlated with UV radiation [17], which is in accordance with the results observed for *F. spiralis* acetone:water (7.3) extracts from both locations (higher values in summer). Salinity is also another parameter considered determinant for brown algal phlorotannin concentrations that increases with increasing salinity in algae habitat [17]. Thus, the significantly higher salinity in SMA in both seasons (see Section 3.2) could have contributed to the highest TPC values found in *F. spiralis* acetone:water (7:3) extracts from this location as compared to the ones from SMG.

According to the literature, brown macroalgae are a wellknown source of phlorotannins; structurally unique polyphenols and highly hydrophilic components that have gathered much attention due to their numerous bioactivities (including strong antioxidant effects) with high commercial interest for pharmaceutical, nutraceutical, cosmetic, and especially food industries [17]. However, due to their polymeric nature and consequently structural complexity, only few reports have focused on the phlorotannins profile from *Fucus* spp. [61]. Furthermore, sparse characterization of individual phlorotannin components has been carried out in *F. spiralis* [15,18,19], although this species is known to produce two types of polymeric phlorotannins of the fucol and fucophlorethol classes endowed with a high antioxidant activity [18].

### 2.9. Total Flavonoid Content (TFC) Determination in F. spiralis Extracts

Although phlorotannins represent the major phenolic constituents in fucaceaen species, as previously mentioned, some authors have also reported the presence of phenolic acids and flavonoids in these algae [17]. It is well known that flavonoids, the largest and most diverse group of phenolic compounds, have a broad spectrum of biological activities, such as strong antioxidants, scavengers of a wide range of reactive oxygen species, and inhibitors of lipid peroxidation [62]. The TFC in *F. spiralis* extracts was quantified as rutin equivalents (RE), and the results are illustrated in Table 3. *F. spiralis* from SMG show higher values of 68.17 and 62 mg RE/g DE for winter and summer methanol extracts, respectively, as compared to the values of 15.33 and 14.20 mg RE/g DE for winter and summer acetone:water (7:3) extracts, respectively. *F. spiralis* from SMA also show higher values of 34.50 and 32 mg RE/g DE for summer and winter methanol extracts, respectively, as compared to the values of 16 and 30.67 mg RE/g DE for summer and winter acetone:water (7:3) extracts, respectively. These results revealed that methanol is more efficient for *F. spiralis* flavonoid extraction as compared to 70% aqueous acetone, and that methanol extracts from SMG summer and winter presented higher TFC values as compared to the other samples under study. The results also revealed that extracts from SMG presented higher values in winter than in summer for both extracts. On the other hand, summer methanol extract from SMA presented higher values as compared to winter, and oppositely, the winter acetone:water (7:3) extract showed higher values than summer.

The TFC values of *F. spiralis* acetone:water (7:3) extracts in the present study were similar to the ones reported by Dang et al. [63] from brown fucoid algae that show the values of 29.31 for *Hormosira banksii*, 22.38 for *Sargassum podacanthum*, and a lower value of 9.89 mg catechin equivalents/g DE for *Phyllospora comosa*, using ethanol:water (7:3) as the extractant. The study of Cox et al. [62] on the TFC of methanol extracts from edible Irish seaweeds also found concentrations in a wide range among the brown algae under study (7.66 to 42.50 mg quercetin equivalents/g DE), presenting the fucoid *Himanthalia elongata* the highest TFC value that was, however, lower than the ones observed in the *F. spiralis* SMG methanol extracts. According to these authors, polyphenols, including tannins and flavonoids, could be mainly responsible for the antioxidant and antimicrobial activities of the algae studied.

### 2.10. Antioxidant Activity Assays in F. spiralis Extracts

#### 2.10.1. 2,2-Diphenyl-1-Picrylhydrazyl (DPPH) Free Radical Scavenging Activity (FRSA) Assay

Assessments of antioxidant properties of natural compounds are very important due to their applications in medicine, food, and cosmetics. The scavenging activity of DPPH free radicals had been extensively used to determine the antioxidant power of bioactive natural products. Table 4 shows the FRSA of the *F. spiralis* extracts obtained as EC_50_ value (mg/mL). *F. spiralis* from SMA show the values of 0.076 and 0.045 mg/mL for methanol and 0.061 and 0.059 mg/mL for acetone:water (7:3) extracts from summer and winter samples, respectively. *F. spiralis* from SMG shows the values of 0.092 and 0.123 mg/mL for methanol and 0.064 and 0.067 mg/mL for acetone:water (7:3) extracts from summer and winter samples, respectively. These results revealed that SMA presented better values of EC_50_ than SMG in both seasons and extracts. In addition, it presents even better values than the BHT (0.062 mg/mL) for the SMA winter season in both extracts. The results are very similar to the reported by Rodrigues [25] that presented the value of EC_50_ of 0.077 mg/mL for a methanol extract of *F. vesiculosus* collected in Aveiro (Portugal). Higher values of EC_50_, that represent lower antioxidant activity, were reported by Tierney et al. [59] for a Irish *F. spiralis* methanol:water extract (0.146 mg/mL) and similar results were also reported for water (0.125 mg/mL) and ethanol:water (0.092 mg/mL) extracts. Another study [64] on the FRSA of dichloromethane:methanol extracts from ten Phaeophyceae algae from Brittany coasts also revealed that the Fucales species (*Bifurcaria bifurcata*, *Cystoseira tamariscifolia*, *Fucus ceranoides*, and *Halidrys siliquosa*) presented the strongest activity, however, showing higher values of EC_50_ (0.21 to 0.56 mg/mL) than the ones found in the *F. spiralis* extracts under study.

The FRSA results of all *F. spiralis* samples show slightly increased values with increasing reaction time (data not shown). Indeed, the knowledge of the kinetics of the DPPH consumption is also important because free radicals in the organism are short-lived species, which implies that the impact of a substance as an antioxidant depends on its fast reactivity towards free radicals [65].

The results of antioxidant properties show that *F. spiralis* extracts, particularly from SMA, are a valuable resource that could be explored from biotechnology and commercial perspectives. The very strong FRSA of *F. spiralis* extracts may be due to its high concentration of phlorotannins, particularly in the acetone:water (7:3) extracts. Our findings are in agreement with those of previous studies on FRSA of extracts/fractions from other algae samples, such as the French and Irish *F. spiralis* [18,19] and other *Fucus* species (*F. vesiculosus*, *F. ceranoides*, and *F. serratus*) [60,64,66]. It should also be highlighted that a recent study by Paiva et al. [16] revealed that high-molecular weight phlorotannins fraction from Azorean *F. spiralis* hydrolysate show a strong antioxidant activity on free radicals and also show high inhibitory activity of ACE-I (angiotensin-I converting enzyme). Another study [20] revealed that phlorotannin-enriched fractions from *F. spiralis* from Peniche coast (Portugal) have the capacity to inhibit in vitro human cellular damage promoted by reactive oxygen species. However, in order to better understand the contribution of the phenolic compounds to the FRSA of *F. spiralis* extracts, its complete chemical characterization should be done in future.

#### 2.10.2. Ferrous Ion-Chelating (FIC) Activity Assay

**Figure 1:**
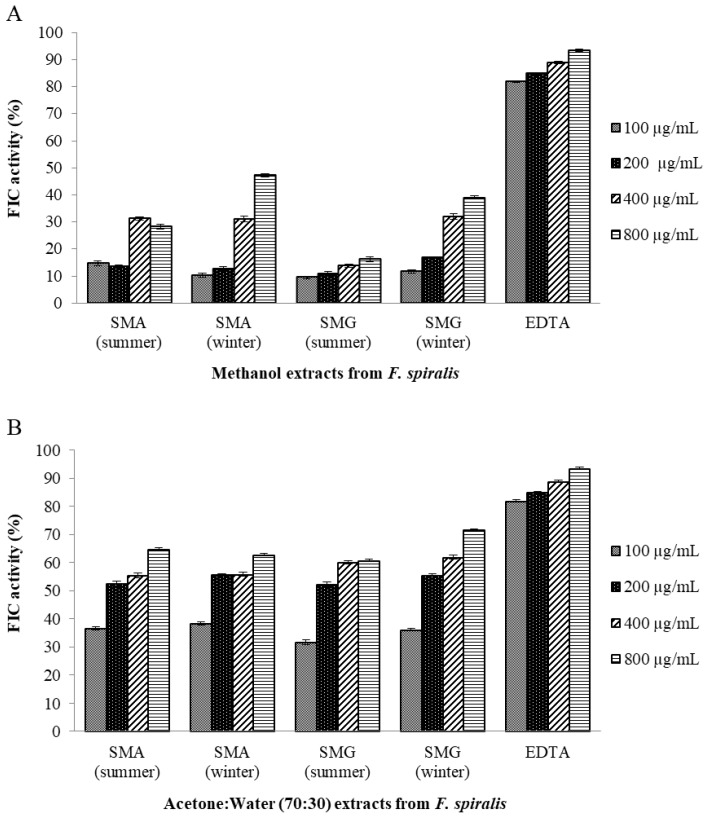
Ferrous ion-chelating (FIC) activities of *F. spiralis* samples. (**A**) *F. spiralis* extracted by methanol. (**B**) *F. spiralis* extracted by acetone:water (70:30). EDTA (ethylenediaminetetra acetic acid) was used as positive control. Values are mean ± SD (*n* = 3). Legend: SMA, samples from Santa Maria Island (Azores); SMG, samples from São Miguel Island (Azores).

Since metal chelating capacity is claimed as one of the important mechanisms of antioxidant activity [67], FIC assay was also chosen to better characterize the antioxidant activity of *F. spiralis* extracts. As shown in Figure 1, SMA and SMG *F. spiralis* from winter methanol extracts presented moderate FIC activity (47.19% and 38.96%, respectively, for the concentration of 800 µg/mL), and *F. spiralis* summer methanol extracts from both islands show lower metal chelating agents with values of 28.25% and 16.12%, respectively, as compared to the synthetic antioxidant EDTA, a potent metal-ion chelator used in this study as a positive control. These results revealed that *F. spiralis* winter methanol extracts presented higher FIC values than from summer in both SMA and SMG islands. Different results were observed with acetone:water (7:3) extracts that show higher FIC activity presenting the values of 60.73% and 64.71% for *F. spiralis* summer extracts of SMG and SMA, respectively, and oppositely, the values of 62.78% and 71.50% for *F. spiralis* winter extracts of SMA and SMG, respectively.

Overall, the results revealed that the FIC activity presents the highest value in acetone:water (7:3) extract of SMG winter, and that the *F. spiralis* FIC activity appears to be dramatically influenced by the solvents used for extraction. Wang et al. [67] reported lower values of 38% for *F. serratus* and 55% for *F. vesiculosus* from the same extract of 70% aqueous acetone. Tierney et al. [59] reported values of 15.51% and 52.74% for ethanol:water and methanol:water extracts from Irish *F. spiralis*, respectively, that are in agreement with our findings.

Some studies have demonstrated that polyphenols derived from brown algae are potent ferrous-ion chelators [68] and that their metal chelating potency is dependent upon their unique phenolic structure and the number and location of the hydroxyl groups [69]. However, to evaluate the better contribution of the polyphenols to the FIC activity of *F. spiralis*, additional research is needed on structural information of the bioactive compounds.

#### 2.10.3. Ferric Reducing Antioxidant Power (FRAP) Assay

The reducing capacity of a compound may serve as a significant indicator of its potential antioxidant activity [70]. Table 4 shows the reducing power of the *F. spiralis* extracts obtained as EC_50_ values (mg/mL). Lower EC_50_ values of FRAP (indicating high FRAP) for *F. spiralis* methanol extracts were observed in SMA winter (0.016 mg/mL) followed by SMG summer (0.019 mg/mL) and SMA summer (0.022 mg/mL), and the highest value was found in SMG winter (0.033 mg/mL). For acetone:water (7:3) extracts, the best value was found in SMA summer (0.017 mg/mL) followed by SMA winter (0.020 mg/mL), and SMG winter and summer samples presented the same value (0.024 mg/mL).

The information on FRAP activity is important because the reducing capacity of *F. spiralis* extracts may serve as a significant indicator of reductones, which are reported to be terminators of free radicals chain reactions, present in the samples [71]. Furthermore, the FRAP assay confirmed, in general terms, the results obtained by the DPPH–FRSA assay. Indeed, Table 4 shows that the FRSA and FRAP values followed a similar pattern in the samples under study, which is explained by the fact that both assays rely on a mechanism of electron/hydrogen donation.

Overall, the results revealed that FRAP shows the best value in the methanol extract of SMA winter and acetone:water extract of SMA summer. As previously mentioned, the methanol extract of SMA winter also presented the highest FRSA, FIC activity, and TPC and the lowest TFC, as compared to the other methanol extract samples. The acetone:water extract of SMA summer presented also the highest TPC values as compared to all other samples under study, as well as a high FRSA and FIC activity. Our findings are in agreement with previous studies on FRAP of extracts from other algae samples, such as the Irish *F. spiralis* [59] and other *Fucus* species (*F. vesiculosus* and *F. serratus*) [60,66], that also found that higher FRAP activity may be attributed to its rich phlorotannins content.

#### 2.10.4. Pearson Correlation between the Antioxidant Activity Parameters

Significant correlations were found to occur among the methods for the antioxidant activities determination in *F. spiralis* extracts from SMA and SMG (winter and summer seasons). Concerning the methanol extracts, FRSA and FIC activity (*r* = 0.988 in winter and 0.944 in summer) were very strongly correlated as well as FIC activity and FRAP (*r* = 0.991 in winter and 0.997 in summer) and between FRSA and FRAP (*r* = 0.997 in winter and 0.940 in summer). Regarding the acetone:water (7:3) extracts the correlation between FRSA and FIC activity were very strong (*r* = 0.930) in the winter season and were moderate correlated (*r* = 0.704) in the summer season. On the other hand, very strong negative correlations were observed between FIC activity and FRAP (*r* = −0.968) in the winter season and were very strongly correlate in the summer season (*r* = 0.921). However, FRSA and FRAP were negative strongly correlated (*r* = −0.821) in the winter season and were moderated (*r* = 0.706) in the summer season.

## 3. Materials and Methods

### 3.1. Chemicals and Reagents

Methanol (MeOH) and chloroform HPLC grade were purchased from Fluka Chemika (Steinheim, Switzerland). Sodium carbonate (Na_2_CO_3_), sodium phosphate, sodium tetraborate decahydrate, sodium hydroxide (NaOH), Kjeldahl catalyst, ethanol, acetone, phenol, acid boric, potassium acetate (KCH_3_CO_2_), potassium hydroxide, and standard glucose were from E. Merck (Darmstadt, Germany). Derivatization reagent (14% boron trifluoride in methanol) was purchased from Alltech Associates (Deerfield, IL, USA). Total Dietary Fiber Assay Kit (TDF-100A Kit) that contains α-amylase (A3306), amyloglucosidase (A9913), protease (P3910) and celite, fatty acids, methyl esters standards “FAME Mix C4-C24 (18919 Supelco)” and “PUFA No. 1 Marine Source (47033 Supelco)”, potassium ferricyanide, iron (II) chloride, iron (III) chloride, ferrozine, trichloroacetic acid (TCA), Folin–Ciocalteu reagent (FCR), phloroglucinol, rutin, butylated hydroxytoluene (BHT), ethylenediaminetetraacetic acid (EDTA), 2,2-diphenyl-1-picrylhydrazyl (DPPH), hydrochloric acid (HCl), aluminum chloride (AlCl_3_), sulphuric acid, hexane, and petroleum ether were obtained from Sigma-Aldrich (St. Louis, MO, USA).

### 3.2. Study Site and Environmental Parameters of Season Collecting Samples

The Azores Archipelago, located in the North Atlantic Ridge, comprises of nine islands distributed into three groups (western, central, and eastern). The selected islands, São Miguel (SMG) and Santa Maria (SMA), belong to the eastern Azorean group. All the islands are of volcanic origin and only in SMA, the most southwestern island of the Azores, are there sedimentary units interbedded in the volcanic succession of basic nature. Although the oldest volcanic rocks are dated at 8.12 million years at this island, a large portion of the archipelago consists of potentially active volcanoes [72].

The Azores Islands are characterized by an oceanic temperate climate, with mild temperatures all year around and well-defined and stable environmental season conditions, but different from season to season. During most of the year (autumn, winter, and early spring), the Azores region is frequently crossed by the North Atlantic storm-track, the main path of rain-producing weather systems. During late spring and summer, the anticyclone influences the Azores climate and there is less rain. The oceanographic environmental conditions are also mostly due to the influence of the Gulf Stream, which transports warm water of equatorial and tropical origin into the colder northern waters. The current patterns result in the high salinity, high temperature, and low nutrient regime that characterizes the Azores seawater [73]*.*

The average seawater temperature during the seasons’ colleting samples in SMG and SMA were 15.6 °C and 22.2 °C for winter and summer in SMG and 16.7 °C and 24.4 °C for winter and summer in SMA. The average rainfall (mm) ranged from 57.8 to 154.0 for summer and winter in SMG and from 18.6 to 107.9 for summer and winter in SMA. The salinity (%) ranged from 32.05 to 35.02 for winter and summer in SMG and from 34.03 to 38.07 for the same seasons in SMA. The total global radiation (RGtot) and density of ultraviolet (DUV) ranged from 17,206.7 to 12,048.3 kJ/m^2^ and 1.808 to 0.299 kJ/m^2^ in SMG for summer and winter, respectively, and ranged from 22,826.2 to 13,686.1 kJ/m^2^ and 2.226 to 0.377 kJ/m^2^ in SMA for summer and winter, respectively [74].

### 3.3. Collection and Preparation of F. spiralis Samples

Specimens of *F. spiralis* Linnaeus (Ochrophyta, Phaeophyceae) were collected on the 22 January 2013 and the 2 September 2014 from the littoral of SMG Island (Ponta Delgada) and on the 30 July 2013 and the 15 February 2014 from the littoral of SMA Island (São Lourenço Bay). Voucher specimens were prepared (AZB, SMG-13-04; SMG-14-70; SMA-13-01; SMA-14-01) and deposited in the Herbarium AZB—Ruy Telles Palhinha of the Department of Biology at the University of Azores. Within 24 h of collection, *F. spiralis* collected samples were first washed in seawater followed by distilled water to remove encrusting material, epiphytes, and salts, and then fast air-dried with paper towels (to reduce oxidative processes) and stored in an air-tight container in a freezer (−80 °C) until further analysis. Prior to the analytical procedures, the samples were defrosted and dried at 40–45 °C for 48 h (avoiding overheating that could lead to oxidation), and then were grounded into a fine powder of 0.5 mm particle size, redried at 40 °C and stored in dark under N_2_ in a desiccator at a refrigerated temperature of 4–5 °C.

### 3.4. Preparation of F. spiralis Extracts

One gram of dried algal powder was defatted with hexane and extracted with 10 mL of methanol or 70% aqueous acetone (*v*/*v*) using a water bath at 70 °C for 2 h. The extraction procedure was repeated three times and the combined extract was centrifuged at 3500 rpm for 10 min and filtered through cellulose acetate membranes (0.45 µm pore size). The filtrate was concentrated to dryness under reduced pressure using a rotary evaporator to remove methanol and acetone and the concentrate was freeze-dried and stored at −80 °C until analyzed. The extraction yield was expressed as g dried extract/100 g dried algal powder.

### 3.5. Nutrient Analysis

#### 3.5.1. Crude Protein Determination

The organic nitrogen content was quantified using the modified Kjeldahl procedure [75] in a Velp Scientifica UDK 132 apparatus [14]. Each of the algal samples (1 g of homogenized dry algal material powder) was digested with 12 mL of sulphuric acid 96%, then distilled with sodium hydroxide (35%) and acid boric solution (2%), and finally titrated with 0.1 M HCl. Estimation of the crude protein content was calculated multiplying the organic nitrogen content by a conversion factor of 6.25 (% Protein = % N × 6.25).

#### 3.5.2. Soluble Carbohydrate Content Determination

The soluble carbohydrate content was extracted from algal samples with 2.5 N HCl and the concentrations determined by the phenol-sulphuric acid colorimetric method described by Dubois et al. [76]. Percent soluble carbohydrate was calculated based on absorption at 490 nm in a Shimadzu 160-A UV/VIS spectrophotometer model 1800 (Shimadzu Co., Kyoto, Japan). The results were calculated from a multiple level glucose calibration curve at five different concentrations, constructed from peak-area versus glucose concentration [14].

#### 3.5.3. Total Lipid and Fatty Acids (FA) Determination

The crude lipid content was determined gravimetrically after soxhlet extraction [77] with chloroform:methanol 2:1 (*v*/*v*) during 4 h of reflux, in order to obtain high-yield, following the Folch et al. [78] methodology. For FA groups determination, a cold extraction with chloroform:methanol (2:1, *v*/*v*) in the absence of light to minimize lipids oxidation was adopted and then the sample was transmethylated using 0.5 N potassium hydroxide methanol solution and derivatized with 14% boron trifluoride in methanol. Fatty acids methyl esters (FAME) were analyzed by GC on a fused silica CP-Wax 58 (FFAP) CB column (25 m × 0.25 mm i.d. and 0.20 µm film thickness) from Varian (Palo Alto, CA, USA), using the analytical conditions previously described by Paiva et al. [14], and for the more difficult assignments, were also confirmed by GC/MS experiments carried out with a Varian 3800 GC (Palo Alto, CA, USA) interfaced with a 4000 Varian MS, using the same analytical conditions. The mass spectrometric conditions were: electron impact ionization at 70 eV, source temperature 220 °C, 100 μA trap current, and the sweep time was 1.5 s/decade scan, with a mass range of 50–450 *m*/*z*. FAME peaks were quantified by area normalization using the workstation software from Bruker Daltonik (Bremen, Germany). Identification of compounds was achieved by comparison of their retention times and mass spectra with those from pure standards injected under the same conditions and from the NIST 05 MS Library Database.

#### 3.5.4. Total, Soluble, and Insoluble Dietary Fiber (TDF, SDF and IDS) Determination

One gram of dried algal sample, previously defatted with petroleum ether, was used for the TDF and IDF determination, according to the AOAC enzymatic gravimetric method [79] and following the Sigma-Aldrich protocol provided in the TDF-100A Kit with modifications as described by Paiva et al. [80]. The SDF was calculated by difference as TDF—IDF [80].

#### 3.5.5. Ash Determination

The total inorganic material (ash) in insoluble indigestible samples (1 g), after water and organic matter have been removed by heating in the presence of oxidizing agents, was determined gravimetrically after incineration of dried algal material at 550 °C for 2 to 3 h using an electric muffle furnace [77]. The ash content was determined using the equation: % Ash = weight of ash/weight of sample × 100.

#### 3.5.6. Energy Value Determination

The energy content of the algal samples was determined following the methodology of Renaud and Luong-Van [58] (a modification of the Brett and Groves [81] method), multiplying the values obtained for protein, soluble carbohydrates, and lipid by 23.86, 17.16, and 36.42 kJ/g, respectively. The protein, soluble carbohydrate, and lipid contents of the algal samples were determined as described in Section 3.5.1, Section 3.5.2 and Section 3.5.3, respectively.

### 3.6. Total Phenolic Content (TPC) Determination in F. spiralis Extracts

TPC was determined according to the method of Waterhouse [82] with slight modifications [16]. An aliquot of 100 µL of each extract samples (2 mg/mL) was mixed with 1500 µL of distilled water and 100 µL of 2N FCR, homogenized in a vortex for 15 s, and placed in dark for 3 min. Then, 300 µL of 10% Na_2_CO_3_ (*w*/*v*) was added to the mixture, homogenized, and incubated for 5 min at 50 °C. The absorbance (Abs) values were measured at 760 nm. A blank sample was prepared by replacing the sample with Milli-Q water. The phloroglucinol (a basic structural unit of phlorotannins) was used as a standard and the results were expressed as mg of phloroglucinol equivalents (PE) per gram of dried extract, and also as mg of PE per gram of dried algal powder. A calibration curve was prepared using a concentration range of 50–300 μg/mL.

### 3.7. Total Flavonoid Content (TFC) Determination in F. spiralis Extracts

TFC was determined according to the method of Chang et al. [83] with slight modifications. An aliquot of 100 µL of each extract sample (2 mg/mL) was mixed with 100 µL of 10% AlCl_3_, 100 µL of 10% KCH_3_CO_2_, and 900 µL of distilled water. The mixture was homogenized in a vortex for 15 s and after 30 min at room temperature, the Abs was determined at 415 nm. The rutin was used as a standard and the results were expressed as mg of rutin equivalents (RE) per gram of dried extract, and also as mg of RE per gram of dried algal powder. A calibration curve was prepared using a concentration range of 12.5–100 μg/mL.

### 3.8. Antioxidant Activity Assays in F. spiralis Extracts

#### 3.8.1. DPPH Free Radical Scavenging Activity (FRSA) Assay

The FRSA of *F. spiralis* samples was determined according to the method of Molyneux [84] with slight modifications [16]. The FRSA of each sample was tested by measuring their ability to quench DPPH. The DPPH, a stable free radical, was reduced changing the purple color of the DPPH radical solution to a bright yellow in the presence of antioxidants that possess hydrogen-donating or chain-breaking properties and the intensity of this can be monitored spectrophotometrically [85]. An aliquot of 250 µL of each extract sample with various concentrations (or BHT) was added to 500 µL of 100 µM DPPH solution. BHT was used as reference sample and a mixture without sample or BHT was used as the control. The Abs was measured at 517 nm after 30 min in the dark. The FRSA was calculated as a percentage of DPPH decoloration using the following equation: % FRSA = (1 − Abs_sample_/Abs_control_) × 100. Above 90% can be considered as a full absorption inhibition of DPPH, because after completing the reaction the final solution always possesses some yellowish color and therefore its absorption inhibition compared to colorless methanol solution cannot reach 100%. Half-maximal effective concentration (EC_50_) was defined as the sample concentration that can quench fifty percent of DPPH free radicals. Lower EC_50_ value (mg/mL) means a higher antioxidant activity.

#### 3.8.2. Ferrous Ion-Chelating (FIC) Activity Assay

Chelating ability of *F. spiralis* samples was determined according to the modified method of Wang et al. [67], by measuring the inhibition of the Fe^2+^–ferrozine complex formation. An aliquot of 100 µL of each extract samples (concentration range 100–800 μg/mL) was mixed with 135 µL of methanol plus 5 µL of 2 mM FeCl_2_. The reaction was initiated by the addition of 10 µL of 5 mM ferrozine. After 10 min at room temperature, the Abs was determined at 562 nm. Methanol instead of ferrozine solution was used as a sample blank, which is used for error correction because of unequal color of the sample solutions. Methanol instead of sample solution was used as a control. Results were expressed as relative iron chelating activity compared with the unchelated (without ferrozine) Fe^2+^ reaction, and EDTA was used as reference standard. A lower Abs indicated a better FIC ability. The FIC ability was calculated as follows: % FIC ability = [A_0_ − (A_1_ − A_2_)]/A_0_ × 100, where A_0_ was the Abs of the control, A_1_ was the Abs of the sample or standard, and A_2_ was the Abs of the blank.

#### 3.8.3. Ferric Reducing Antioxidant Power (FRAP) Assay

The FRAP of *F. spiralis* samples was determined according to the method of Oyaizu [86], and evaluated on the basis of their abilities to reduce Fe^3+^ complex to Fe^2+^. An increased Abs value indicates an increased reducing power of the algal extract sample. Each extract sample (concentration range 12.5–100 μg/mL) in methanol (0.4 mL) was mixed with 0.4 mL of 200 mM of phosphate buffer (pH 6.6) plus 0.4 mL of potassium ferricyanide (1%, *w*/*v*) and the mixture was incubated at 50 °C for 20 min. After cooling down, 0.4 mL of TCA (10%, *w*/*v*) was added, and the mixture was centrifuged at 3000× *g* for 10 min. The upper layer (1 mL) was mixed with 1 mL of deionized water plus 0.2 mL of FeCl_3_ (0.1% *w*/*v*), and the Abs was measured at 700 nm against a blank. The blank solution contained pure methanol instead of the methanolic extract sample. BHT was used for comparison. EC_50_ value (mg/mL) is the effective concentration at which the absorbance was 0.5 for reducing power and was obtained by interpolation from linear regression analysis of concentration vs. absorbance at 700 nm. A lower EC_50_ value means a higher antioxidant activity.

### 3.9. Statistical Analysis

All determinations were performed at least in triplicate and the results were expressed as means ± standard deviations (SD). The statistics analysis was performed using SPSS 17.0 (version 17, SPSS Inc., Chicago, IL, USA) and one-way analysis of variance test (ANOVA) was carried out to assess for any significant differences between the means. Differences between means at the 5% (*p* < 0.05) level were considered significant. Correlations between the antioxidant activities parameters were obtained using Pearson’s correlation coefficient (*r*).

## 4. Conclusions

Limited published data is available on the effect of harvest time and geographical origin on the metabolites composition and biological activities of edible *Fucus* species, despite the fact they can provide key information in relation to their future exploitation as sustainable sources of functional food ingredients. To the best of our knowledge, this is the first study that reports the seasonal variability (winter and summer) of the biochemical composition and antioxidant properties of *F. spiralis* at two different Azorean islands (São Miguel–SMG and Santa Maria–SMA).

Concerning the biochemical composition of *F. spiralis* samples, protein content is higher in SMG than in SMA with best results in winter, and TDF, the most abundant component, oppositely presents the maximum values in SMA than in SMG with higher values in summer. Ash, the second most abundant component, is higher in SMA winter, and soluble carbohydrates are higher in SMG winter and SMA summer. The energy value is higher in SMA summer which also presents significantly higher lipid content. MUFA content is remarkably higher in summer, the PUFA content is maximum in SMG winter, which also presents an excellent *n*6/*n*3 ratio, and h/H ratio is maximum in SMG summer.

Regarding the antioxidant properties of *F. spiralis* extracts, TPC is higher in acetone:water (7:3) than methanol extracts with best values in SMA. Oppositely, TFC is higher in methanol extracts with the best values in SMG. FRSA (EC_50_) presents the best values in SMA winter in both extracts, showing even better values than the BHT assessed under the same assay conditions. The FIC activity (%) presents the highest value in acetone:water (7:3) extract of SMG winter, and FRAP (EC_50_) shows the best values in methanol extract of SMA winter and acetone:water (7:3) extract of SMA summer. The results suggest that Azorean *F. spiralis* extracts are endowed with a great potential for the development of novel antioxidant products.

The present study shows the influence of geographical origin, seasonal conditions, and probably, the effect of the different geological composition of the coastal rocks of SMA (that are millions of years older than SMG) on *F. spiralis* biochemical composition and antioxidant properties, providing useful information of the best location and period of the year to collect this seaweed for a specific use. However, further studies will need in order to understand the complex mechanisms controlling the *F. spiralis* metabolism plasticity. Namely, future laboratory experiments are required in order to better understand the impact of single and synergistic effects of environmental factors on the observed variability.

The present study also shows that, taking into account the low pollution level of the Azorean seawater and the nutritional and functional bioactivity values of *F. spiralis* (high TDF and ash contents, balanced SDF/IDF, *n*6/*n*3 and h/H ratios, and a great antioxidant potential), the regular consumption of this seaweed can have human health benefits, particularly in the Western countries. Additionally, *F. spiralis* may also be potentially profitable from biotechnology and commercial perspectives with some impact on the economy of the Azores Islands, which in turn can provide an incentive for the biodiversity conservation and for the maintenance of a clean environment in the Azores Archipelago. However, biological evaluation using human and animal feeding studies would be required to corroborate the nutritional and functional food values of *F. spiralis* obtained here based on chemical analyses.

## Figures and Tables

**Table 1:** Composition (protein, lipids, soluble carbohydrates, ash, and total, soluble, and insoluble dietary fiber) and calculated energy value of *F. spiralis* samples ^*a*^.

Composition and Energy Value	*F. spiralis* Samples			
	SMA (Summer)	SMA (Winter)	SMG (Summer)	SMG (Winter) ^*d*^
Protein ^*b*^	4.14 ± 0.10 ^d^	6.85 ± 0.10 ^c^	8.25± 0.13 ^b^	9.71 ± 0.03 ^a^
Lipids ^*b*^	11.54 ± 0.51 ^a^	4.40 ± 0.10 ^b^	5.33 ± 0.31 ^b^	5.23 ± 0.03 ^b^
Carbohydrates ^*b*^	17.03 ± 0.59 ^a^	12.77 ± 0.65 ^b^	13.45 ± 0.79 ^b^	17.59 ± 0.27 ^a^
Ash ^*b*^	22.43 ± 0.45 ^c^	29.57 ± 0.55 ^a^	25.40 ± 0.36 ^b^	22.67 ± 0.47 ^c^
TDF ^*b*^	52.27 ± 1.53 ^a^	50.24 ± 1.40 ^a^	40.47 ± 1.33 ^b^	40.44 ± 0.61 ^b^
SDF ^*b*^	24.77 ± 2.07 ^a^	24.71 ± 1.53 ^a^	17.77 ± 2.26 ^bc^	18.75 ± 0.97 ^b^
IDF ^*b*^	27.50 ± 0.61 ^a^	25.53 ± 0.77 ^b^	22.70 ± 1.01 ^c^	21.69 ± 0.97 ^c^
SDF/IDF ratio	0.90	0.97	0.78	0.86
Energy value ^*c*^	8.12 ± 0.25 ^a^	5.43 ± 0.14 ^d^	6.22 ± 0.04 ^c^	7.24 ± 0.05 ^b^

^*a*^ Values are mean ± SD (*n* = 3). Different superscript letters are significantly different (*p* < 0.05). ^*b*^ Percentage of dry weight. ^*c*^ kJ/g of dry weight. ^*d*^ The protein, lipids, and carbohydrates contents were previously referred by the authors [14]. TDF, total dietary fiber; SDF, soluble dietary fiber; IDF, insoluble dietary fiber. The SDF was calculated by difference as TDF—IDF. SMA, samples from Santa Maria Island (Azores). SMG, samples from São Miguel Island (Azores).

**Table 2:** Gas chromatography determination of fatty acid composition (% of total FAME) of *F. spiralis* samples.

Fatty Acids Profile and Fatty Acids Groups	RT of FAME (min)	*F. spiralis* Samples			
		SMA (Summer)	SMA (Winter)	SMG (Summer)	SMG (Winter)
**Fatty Acids**					
Myristic, C14:0	5.75	14.75 ± 1.26	13.59 ± 0.98	13.73 ± 0.92	11.35 ± 0.90
Myristoleic, C14:1 *c*9 (*n*5)	6.271	tc	tc	0.54 ± 0.09	1.25 ± 0.15
Palmitic, C16:0	8.903	19.49 ± 1.21	23.62 ± 1.43	15.34 ± 1.02	20.15 ± 1.61
Palmitoleic, C16:1 *c*9 (*n*7)	9.309	tc	tc	1.21 ± 0.09	1.06 ± 0.08
Heptadecenoic, C17:1 *c*10 (*n*7)	11.087	tc	0	0.46 ± 0.05	1.39 ± 0.10
Stearic, C18:0	12.487	1.20 ± 0.11	3.44 ± 0.39	1.57 ± 0.15	1.01 ± 0.04
Oleic, C18:1 *c*9 (*n*9)	12.845	29.72 ± 2.41	21.62 ± 1.11	38.04 ± 1.91	20.36 ± 1.43
*Cis*-7-Octadecenoic, C18:1 *c*7 (*n*11)	12.939	tc	0	tc	tc
Linolelaidic, C18:2 *t*9,12 (*n*6)	13.666	5.27 ± 0.74	4.35 ± 0.34	5.24 ± 0.39	6.15 ± 0.57
Linoleic (LA), C18:2 *c*9,12 (*n*6)	13.759	tc	tc	0.45 ± 0.08	tc
Arachidic, C20:0	14.233	tc	0	tc	tc
γ-Linolenic (GLA), C18:3 *c*6,9,12 (*n*6)	14.868	3.20 ± 0.51	6.73 ± 0.98	3.46 ± 0.80	7.04 ± 0.81
Eicosenoic, C20:1 *c*11 (*n*9)	16,196	tc	tc	0.57 ± 0.14	tc
α-Linolenic (ALA), C18:3 *c*9,12,15 (*n*3)	16.516	tc	tc	tc	tc
Heneicosanoic, C21:0	17.381	tc	tc	0.37 ± 0.06	tc
Eicosadienoic, C20:2 *c*11,14 (*n*6)	17.871	tc	tc	tc	tc
Dihomo-γ-linolenic (DHGLA), C20:3 *c*8,11,14 (*n*6)	18.289	11.96 ± 1.10	9.40 ± 1.00	11.87 ± 0.98	13.83 ± 1.10
Eicosatrienoic, C20:3 *c*11,14,17 (*n*3)	19.484	5.32 ± 0.41	7.80 ± 0.33	4.33 ± 0.39	11.31 ± 0.95
Arachidonic (ARA), C20:4 *c*5,8,11,14 (*n*6)	19.812	tc	tc	tc	tc
Docosadienoic, C22:2 *c*13,16 (*n*6)	20.991	1.90 ± 0.22	2.20 ± 0.20	tc	tc
Lignoceric, C24:0	21.526	tc	0	tc	0
Eicosapentaenoic (EPA), C20:5 *c*5,8,11,14,17 (*n*3)	23.263	1.89 ± 0.29	3.44 ± 0.24	0.62 ± 0.09	1.01 ± 0.07
Nervonic, C24:1 *c*15 (*n*9)	23.579	3.82 ± 0.31	tc	tc	2.83 ± 0.20
Docosahexaenoic (DHA), C22:6 *c*4,7,10,13,16,19 (*n*3)	35.159	1.49 ± 0.23	3.80 ± 1.06	2.20 ± 0.76	1.25 ± 0.09
**Fatty Acids Groups**					
Total saturated fatty acids (SFA)	-	35.44 ± 1.64	40.65 ± 1.49	31.01 ± 1.83	32.51 ± 1.64
Total monounsaturated fatty acids (MUFA)	-	33.54 ± 1.98	21.62 ± 1.11	40.82 ± 2.08	26.89 ± 1.46
Total polyunsaturated fatty acids (PUFA)	-	31.03 ± 1.34	37.72 ± 1.57	28.17 ± 1.08	40.59 ± 2.11
Total trans fatty acids (TFA)	-	5.27 ± 0.74	4.35 ± 0.34	5.24 ± 0.39	6.15 ± 0.57
Total *n*3 fatty acids	-	8.70 ± 0.41	15.04 ± 1.29	7.15 ± 0.70	13.57 ± 0.83
Total *n*6 fatty acids	-	22.33 ± 1.21	22.68 ± 1.12	21.02 ± 1.99	27.02 ± 1.50
Total *n*9 fatty acids	-	33.54 ± 1.98	21.62 ± 1.11	38.61 ± 1.94	23.19 ± 1.28
Ratio *n*6/*n*3	-	2.57	1.51	2.94	1.99
Ratio h/H	-	1.89	1.59	2.37	2.14

Values are mean ± SD (*n* = 3). tc, trace; 0, compound not detected in sample; RT, retention time; FAME, fatty acids methyl esters; *c*, cis; *t*, trans; *n*, omega; *n*6/*n*3, omega 6 to omega 3 PUFA ratio; h/H, hypocholesterolemic (MUFA + PUFA) to hypercholesterolemic (C14:0 + C16:0) FA ratio. SMA, samples from Santa Maria Island (Azores). SMG, samples from São Miguel Island (Azores). Identification of the individual FAME was achieved by comparison of their RT and mass spectra with those from pure standards injected under the same analytical conditions.

**Table 3:** Yield, total phenolic content (TPC) and total flavonoid content (TFC) in methanolic and acetone:water (70:30) dry extracts of *F. spiralis* samples. TPC and TFC values were expressed as mg per g of extract and also as mg per g of algal powder.

*F. spiralis* Samples	Yield (%)	TPC		TFC	
		mg PE/g DW	mg PE/g DE	mg RE/g DW	mg RE/g DE
**Methanolic Extracts**					
SMA (summer)	11.90	19.21 ± 0.28 ^a^	172.00 ± 3.00 ^a^	4.21 ± 0.16 ^b^	34.50 ± 1.32 ^b^
SMA (winter)	10.30	20.42 ± 0.33 ^a^	187.00 ± 2.65 ^a^	4.10 ± 0.09 ^b^	32.00 ± 0.87 ^b^
SMG (summer)	9.70	10.95 ± 0.20 ^c^	113.33 ± 2.08 ^c^	6.02 ± 0.08 ^a^	62.00 ± 0.87 ^a^
SMG (winter)	9.90	15.18 ± 0.06 ^b^	153.33 ± 0.58 ^b^	6.75 ± 0.03 ^a^	68.17 ± 0.29 ^a^
**Acetone:Water Extracts (70:30)**					
SMA (summer)	19.00	46.60 ± 1.15 ^a^	245.67 ± 5.86 ^a^	3.04 ± 0.10 ^b^	16.00 ± 0.50 ^b^
SMA (winter)	14.30	34.77 ± 0.58 ^b^	243.33 ± 3.79 ^a^	4.09 ± 0.11 ^a^	30.67 ± 0.76 ^a^
SMG (summer)	13.00	29.78 ± 0.75 ^c^	229.33 ± 5.86 ^b^	1.84 ± 0.10 ^c^	14.20 ± 0.72 ^b^
SMG (winter)	14.90	25.27 ± 0.51 ^d^	170.67 ± 3.06 ^c^	2.28 ± 0.11 ^c^	15.33 ± 0.76 ^b^

Values are mean ± SD (*n* = 3). Different superscript letters are significantly different (*p* < 0.05). Tested concentration for TPC and TFC assays = 2 mg/mL. DW, dry weight of algal powder; DE, dry extract; PE, phloroglucinol equivalents; RE, rutin equivalents. SMA, samples from Santa Maria Island (Azores). SMG, samples from São Miguel Island (Azores).

**Table 4:** Free radical scavenging activity (FRSA) and ferric reducing antioxidant power (FRAP) in methanolic and acetone:water (70:30) dry extracts of *F. spiralis* samples, and comparison of the antioxidant activities with BHT ^*a*^.

*F. spiralis* Samples and BHT	FRSA (EC_50_^*b*^, mg/mL)	FRAP (EC_50_^*c*^, mg/mL)
**Methanolic Extracts**		
SMA (summer)	0.076 ± 0.005 ^c^	0.022 ± 0.006 ^b^
SMA (winter)	0.045 ± 0.004 ^d^	0.016 ± 0.004 ^cd^
SMG (summer)	0.092 ± 0.005 ^b^	0.019 ± 0.004 ^bc^
SMG (winter)	0.123 ± 0.01 ^a^	0.033 ± 0.005 ^a^
**Acetone:Water Extracts (70:30)**		
SMA (summer)	0.061 ± 0.004 ^cd^	0.017 ± 0.004 ^c^
SMA (winter)	0.059 ± 0.004 ^e^	0.020 ± 0.006 ^b^
SMG (summer)	0.064 ± 0.007 ^b^	0.024 ± 0.005 ^a^
SMG (winter)	0.067 ± 0.006 ^a^	0.024 ± 0.005 ^a^
BHT	0.062 ± 0.006 ^c^	0.006 ± 0.001 ^d^

^*a*^ Values are mean ± SD (*n* = 3). Different superscript letters are significantly different (*p* < 0.05). ^*b*^ The half maximal effect concentration. ^*c*^ The effective concentration at which the absorbance is 0.5. BHT, butylated hydroxytoluene. SMA, samples from Santa Maria Island (Azores). SMG, samples from São Miguel Island (Azores).
